# Collapsed methylation quantitative trait loci analysis for low frequency and rare variants

**DOI:** 10.1093/hmg/ddw283

**Published:** 2016-08-24

**Authors:** Tom G. Richardson, Hashem A. Shihab, Gibran Hemani, Jie Zheng, Eilis Hannon, Jonathan Mill, Elena Carnero-Montoro, Jordana T. Bell, Oliver Lyttleton, Wendy L. McArdle, Susan M. Ring, Santiago Rodriguez, Colin Campbell, George Davey Smith, Caroline L. Relton, Nicholas J. Timpson, Tom R. Gaunt

**Affiliations:** 1 MRC Integrative Epidemiology Unit (IEU), School of Social and Community Medicine, University of Bristol, Oakfield House, Oakfield Grove, Bristol, UK; 2 University of Exeter Medical School, University of Exeter, Exeter, UK; 3 Institute of Psychiatry, King’s College London, London, UK; 4 Department of Twin Research and Genetic Epidemiology, King’s College London, London, UK; 5 Avon Longitudinal Study of Parents and Children (ALSPAC) & School of Social and Community Medicine, University of Bristol, Bristol, UK; 6 Bristol Genetic Epidemiology Laboratories, School of Social and Community Medicine, University of Bristol, Bristol, UK; 7 Intelligent Systems Laboratory, University of Bristol, Bristol, UK

## Abstract

**Background:** Single variant approaches have been successful in identifying DNA methylation quantitative trait loci (mQTL), although as with complex traits they lack the statistical power to identify the effects from rare genetic variants. We have undertaken extensive analyses to identify regions of low frequency and rare variants that are associated with DNA methylation levels.

**Methods:** We used repeated measurements of DNA methylation from five different life stages in human blood, taken from the Avon Longitudinal Study of Parents and Children (ALSPAC) cohort. Variants were collapsed across CpG islands and their flanking regions to identify variants collectively associated with methylation, where no single variant was individually responsible for the observed signal. All analyses were undertaken using the sequence kernel association test.

**Results:** For loci where no individual variant mQTL was observed based on a single variant analysis, we identified 95 unique regions where the combined effect of low frequency variants (MAF ≤ 5%) provided strong evidence of association with methylation. For loci where there was previous evidence of an individual variant mQTL, a further 3 regions provided evidence of association between multiple low frequency variants and methylation levels. Effects were observed consistently across 5 different time points in the lifecourse and evidence of replication in the TwinsUK and Exeter cohorts was also identified.

**Conclusion:** We have demonstrated the potential of this novel approach to mQTL analysis by analysing the combined effect of multiple low frequency or rare variants. Future studies should benefit from applying this approach as a complementary follow up to single variant analyses.

## Introduction

Genome wide association studies (GWAS) have had a profound influence on the number of complex diseases associated common variants identified. Current endeavours have now shifted to elucidate the functional role of these variants and to better understand the underlying mechanisms by which they influence phenotypic varition. One approach to this has been to determine their impact on DNA methylation, an epigenetic regulation mechanism known to play a key role in many biological processes and disease susceptibility (1,2). Recent studies have found success in identifying methylation quantitative trait loci (mQTLs) using individual variant approaches (3–5). However, these approaches have limited power to detect effects from rare variants, which is also true when analysing complex traits. However, there may be many low frequency and rare variants across the genome which can help explain a large proportion of the additive genetic variance of complex traits and diseases (6,7).

Collaborative efforts have found success in improving the statistical power to detect disease associated rare variants by pooling large sample sizes together (termed meta-GWAS) (8,9). However, adopting such an approach to uncover mQTL caused by rare variation is challenging for several reasons, such as measurements being taken from differing tissue types, samples with a wide range of disease states and matching studies which have defined different quality control parameters. An alternative and feasible approach to leverage statistical power for rare variant analysis involves collapsing them together across the same functional unit or genomic region and analysing their combined effect on phenotypic traits (10,11).

We have undertaken extensive analyses using repeated measures of methylation data from the Accessible Resource for Integrated Epigenomic Studies (ARIES)(12) project to identify mQTL effects from collapsed regions of low frequency and rare variants. Our sample consisted of mother-offspring pairs from the Avon Longitudinal Study of Parents and Children (ALSPAC)(13,14) cohort. Our aim was to identify regions surrounding CpG islands where no single variant was sufficiently responsible for the observed association signal, but rather a combined effect contributed to by several variants not detected by a single variant analysis. Moreover, for CpG islands where there was evidence of a single variant mQTL, we wanted to investigate these regions to evaluate whether there were any independent effects from low frequency and rare variants.

## Results

All analyses were undertaken using the ARIES dataset (12) which includes 450k DNA methylation data collected at five different time points across the life course using individuals from the ALSPAC cohort (13,14). Study characteristics for data from ARIES can be located in Table 1. The childhood time point in ARIES (*n =* 834, Mean Age = 7.49 (Standard Deviation = 0.15), proportion female = 0.50) was selected as the discovery analysis for this study and all results are from this data unless stated otherwise. The imputed genotype dataset for these analyses contained 3,721,682 low frequency variants (MAF ≤ 5%). 1,787,681 of these were rare variants (MAF ≤ 1%).

**Table 1.** Study characteristics

Time point	Sample Size	Mean Age	Proportion Female
**Birth**	771	NA (all zero)	0.49
**Childhood**	834	7.49 (0.15)	0.50
**Adolescence**	837	17.14 (1.01)	0.49
**Pregnancy**	764	29.22 (4.41)	1 (all female)
**Middle Age**	742	47.45 (4.46)	1 (all female)

-Study characteristics for ALSPAC individuals enrolled in the ARIES project across five different life stages in human blood.

### Discovering novel mQTL

There were 27,176 CpG island regions according to UCSC annotations using the hg19 build of the human reference genome(15). 10,836 of these regions had a reported mQTL in close proximity (i.e. within the island or its flanking regions ± 1kb). This was based on the results of a single variant analysis previously undertaken in the ARIES dataset by Gaunt *et al*. (16). Of the remaining 16,340 CpG islands, variants were first of all collapsed across just CpG islands themselves, followed by expanded regions of interest to also include flanking shore and shelf regions. The following numbers of regions had at least 2 low frequency or rare variants within them and were therefore eligible for analysis:

1. 2,934 CpG islands with no flanking regions
2. A further 8,701 CpG islands with shore regions (i.e. islands +2kbs up and downstream)
3. A further 4,407 CpG islands with shore and shelf regions (i.e. islands +4kbs up and downstream).

**Figure 1.**
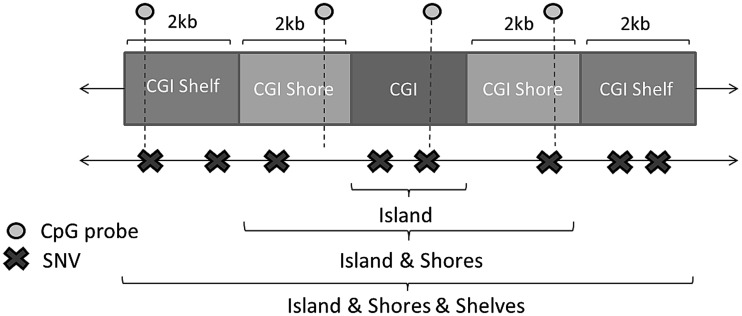
A simple diagram of a theoretical CpG Island and flanking regions. These regions of interest were proposed to aggregate variants together over and analyse their combined effect on measures of methylation at CpG probes.

An illustration of these 3 definitions can be found in Figure 1.

#### Cis-mQTLs

Each collapsed region of variants was analysed in turn with each good quality CpG probe (294,905 out of 485,577, based on evaluations by Naeem *et al*. (17)) within 1 mega base (MB) distance of the region analysed to identify cis-effects. The Sequence Kernel association Test (SKAT) (18) was used in all analyses to evaluate associations between sets of variants and methylation, using two MAF cut offs of ≤ 5% and ≤ 1%. For consistency, we applied the same p-value threshold as Gaunt *et al.* (19) when evaluating findings in the subsequent analyses (*P* < 1.0 × 10 ^−^ ^14^).

**Figure 2.**
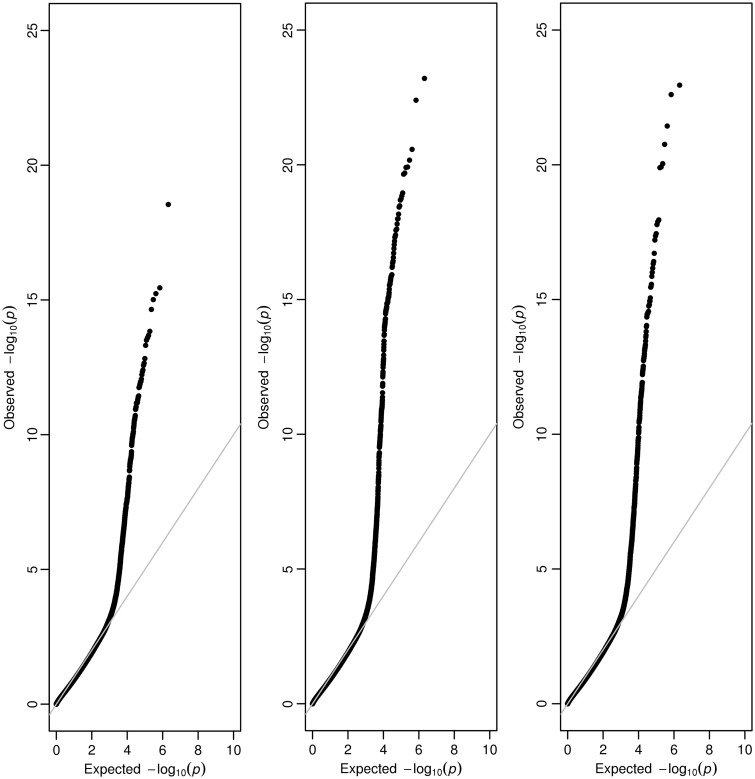
Quantile-Quantile plots for results of the cis-mQTL analysis after aggregating variants according to 1) CpG islands 2) CpG islands & shores 3) CpG islands, shores and shelves.

Methylation levels at five positions were influenced by low frequency variants (MAF ≤ 5%) that were restricted to being located within proximal CpG islands (Supplementary Material, Table S1). Extending these regions to include variants within adjacent shores provided strong evidence of association for 90 unique regions, 88 of which were not identified when collapsing variants from island regions alone. The top hits for these results can be located in Table 2. Extending our region of interest out further to include islands along with shores and shelves identified 37 unique regions with strong evidence of association for cis-mQTL, although only one of these regions was not previously identified using island and shore regions alone in the previous analyses (Supplementary Material, Table S2). Q-Q plots for all these results can be found in Figure 2. Using a MAF threshold of ≤ 1%, we only observed strong evidence of association between one region of variants (chromosome 2: 233,243,999-233,248,448 (CpG island & shores)) and nearby CpG probe cg16700265, near *ALPP* (*P =* 3.62 × 10^−17^). Results can be found in Supplementary Materials, Table S3–S5.

**Table 2.** Analysis of variants within CpG islands & shores (MAF ≤ 5%)

CpG Island & Shores	Nearest Gene	Probe	nVars	*P*-value
chr21:45,728,220–45,732,444	*PFKL*	cg21069494	6	6.24x10^−24^
chr8:144,715,866–144,720,798	*ZNF623*	cg16316162	3	4.02x10^−23^
chr11:66,492,937–66,498,387	*SPTBN2*	cg24851651	6	2.68x10^−21^
chr2:75,785,717–75,790,312	*EVA1A*	cg26175789	8	6.73x10^−21^
chr12:120,753,346–120,757,672	*PLA2G1B*	cg06379361	5	1.21x10^−20^
chr7:100,873,555–100,878,212	*CLDN15*	cg01299997	7	1.26x10^−20^
chr3:53,076,956–53,083,101	*RFT1*	cg04865290	10	2.03x10^−20^
chr2:196,519,555–196,524,950	*SLC39A10*	cg19655195	8	2.23x10^−20^
chr1:1,287,707–1,293,126	*MXRA8*	cg17132079	3	1.11x10^−19^
chr11:64,407,877–64,413,253	*NRXN2*	cg19395706	4	1.43x10^−19^

Top 10 results for analysis between low frequency variants collapsed within CpG Island & flanking Shore regions (where there is no previously reported single variant mQTL effect) and nearby methylation probe (+/- 1Mb of region analysed). nVars = number of variants analysed. Probe = 450k probe ID. *P*-value = *P*-value according to SKAT.

All sets of variants responsible for cis-mQTL effects that survived the correction for multiple comparisons were further evaluated by calculating individual variant effects using linear regression. This was to ensure that no single variant was independently responsible for evidence of association, as well as discerning which variants were collectively responsible for driving the observed signal (Supplementary Materials, Tables S6–S11).

#### Trans-mQTLs

 2,553,242,326 analyses were undertaken to evaluate all possible trans-mQTL using CpG islands and flanking shore regions ((8,701 regions x 294,905 probes) subtracting 12,726,079 possible cis-mQTL effects). Due to the computational burden of this analysis, only CpG island and shore regions were evaluated as this region of interest appeared to be the most effective at identifying evidence of cis-mQTL in the previous analysis (88 of the 94 cis-mQTL effects were observed using this region of interest).

Only one result survived the conservative multiple testing correction. The observed association was found between 9 low frequency variants (MAF ≤ 5%) located on chromosome 21 (between 33,982,367 – 33,987,450, near *C21orf5)* and cg09050820 (chromosome 6:167,586,206 near *TCP10L2*) with a *P*-value of 1.50 × 10^−15^. No observed associations survived the multiple testing correction using a cutoff of 1% MAF. Results can be found in Supplementary Materials, Tables S12 and S13.

### Analyses in other time points

All results that survived the correction for multiple testing were further evaluated using all 4 other time points in the ARIES project which included offspring previously analysed as well as their mothers. Using the top 10 hits of the CpG island and shore analysis, which provided the strongest evidence of association for novel mQTL, we observed consistently low p-values across all other time points in ARIES. These results can be located in Table 3. Effects across other time points for all other hits can be located in Supplementary Materials, Tables S14 and S15.

**Table 3.** Evaluations using other time points in ARIES

CpG Island & Shores	Probe	**Offspring**		**Mothers**	
		Birth (*n = *771)	**Adolescence (***n = ***837)**	**Pregnancy (***n = ***764)**	**Middle Age (***n = ***742)**
chr21:45,728,220–45,732,444	cg21069494	9.34x10^−20^	1.63x10^−18^	2.15x10^−18^	2.39x10^−20^
chr8:144,715,866–144,720,798	cg16316162	5.72x10^−22^	2.62x10^−19^	1.29x10^−23^	3.73x10^−28^
chr11:66,492,937–66,498,387	cg24851651	1.06x10^−12^	7.04x10^−17^	4.52x10^−12^	1.76x10^−11^
chr2:75,785,717–75,790,312	cg26175789	3.27x10^−14^	1.08x10^−20^	7.24x10^−19^	5.47x10^−18^
chr12:120,753,346–120,757,672	cg06379361	3.99x10^−20^	8.86x10^−16^	3.66x10^−19^	6.11x10^−21^
chr7:100,873,555–100,878,212	cg01299997	4.08x10^−22^	3.75x10^−15^	1.96x10^−12^	3.33x10^−19^
chr3:53,076,956–53,083,101	cg04865290	1.34x10^−12^	6.69x10^−24^	3.82x10^−22^	1.99x10^−13^
chr2:196,519,555–196,524,950	cg19655195	1.25x10^−14^	1.68x10^−18^	1.84x10^−15^	6.86x10^−16^
chr1:1,287,707–1,293,126	cg17132079	2.99x10^−13^	4.02x10^−20^	1.06x10^−14^	4.54x10^−14^
chr11:64,407,877–64,413,253	cg19395706	7.53x10^−14^	3.00x10^−16^	5.63x10^−17^	7.56x10^−23^

Each column refers to a different time point within ARIES than the one used in the discovery analysis, as described in Table 1. All columns contain p-values according to SKAT. Probe = 450k probe ID, *n =* sample size at each time point.

### Replication in independent cohorts

Evidence of replication for the top 10 hits in the CpG island and shore analysis was evaluated using individuals from two independent cohorts, TwinsUK (*n =* 847) (20) and Exeter (*n =* 608) (Hannon *et al.*, manuscript in preparation). Replication analyses were successful as low p-values were observed for each mQTL (*P < *0.01). Results can be found in Table 4.

**Table 4.** Replication analysis in the TwinsUK and Exeter cohorts

CpG Island & Shores	Probe	**ALSPAC**		**TwinsUK**		**Exeter**	
		nVars	P-value	nVars	P-value	nVars	P-value
chr21:45,728,220–45,732,444	cg21069494	6	6.24x10^−24^	6	8.13x10^−5^	0	N/A
chr8:144,715,866–144,720,798	cg16316162	3	4.02x10^−23^	3	1.14x10^−6^	2	4.67x10^−18^
chr11:66,492,937–66,498,387	cg24851651	6	2.68x10^−21^	6	0.03	2	2.14x10^−4^
chr2:75,785,717–75,790,312	cg26175789	8	6.73x10^−21^	8	1.42x10^−3^	5	7.57x10^−16^
chr12:120,753,346–120,757,672	cg06379361	5	1.21x10^−20^	5	8.23x10^−3^	2	1.63x10^−7^
chr7:100,873,555–100,878,212	cg01299997	7	1.26x10^−20^	7	4.85x10^−5^	3	8.45x10^−3^
chr3:53,076,956–53,083,101	cg04865290	10	2.03x10^−20^	10	9.23x10^−6^	3	5.44x10^−18^
chr2:196,519,555–196,524,950	cg19655195	8	2.23x10^−20^	8	4.55x10^−3^	3	1.85x10^−7^
chr1:1,287,707–1,293,126	cg17132079	3	1.11x10^−19^	3	1.01x10^−3^	0	N/A
chr11:64,407,877–64,413,253	cg19395706	4	1.43x10^−19^	4	7.61x10^−7^	0	N/A

nVars = number of variants analysed. This varied depending on imputation quality for each cohort. *P*-value according to SKAT.

### Leave-one-out analysis

To ensure that our results were robust to outliers (i.e. individual low frequency/rare variants associated with extremely high/low methylation), we firstly generated box and whisker plots to visually inspect possible outliers for the top 10 hits of the CpG island and shore analysis (Supplementary Material, Fig. S1). These figures did not suggest outliers were an issue in our analysis, although to be certain we have also undertaken leave one out analyses to discern whether signals were driven by a single variant influenced by extreme measurements of methylation. Variants within regions were firstly pruned using plink software (21), followed by re-running our analysis for each mQTL except systematically removing individual variants in turn. Results were plotted with –log10 p-values on the y-axis and the variant removed on the x-axis. Overall, these results help illustrate that collapsed mQTL are a combined effect of low frequency and rare variants on DNA methylation, where evidence of association remains consistently strong even when removing each variant in turn. Furthermore, these results show that our approach is robust to the presence of outliers and linkage disequilibrium between variants. The plots for these analyses can be located in Supplementary Material, Figure S2.

### Whole genome sequence evaluation

To verify that observed associations were not due to an overlapping rare variant in the probe sequence, we identified a subset of 394 individuals from the discovery analysis who had whole genome sequence (WGS) data as part of the UK10K project (22). For the top 10 hits found in Table 2 from our discovery analysis, only 1 probe had an overlapping rare variant based on the UK10K WGS data (cg17132079). We therefore repeated the analysis of the 3 low frequency variants at the CpG island near *MXRA8* and this probe. The observed p-value had attenuated due to the reduction in sample size (*P =* 1.62×10^−11^). However, after conditioning our analysis by including the overlapping rare variant as a covariate in our model we did not observe an attenuation in the observe effect (*P =* 1.38×10^−11^), suggesting that this analysis was not biased by the overlapping rare variant.

### Conditional mQTL analysis

For the remaining 10,836 CpG island regions not evaluated in the initial mQTL analysis, 2,433 had two or more variants within their island or flanking shore region. These regions were not previously analysed due to evidence of mQTL detected in close proximity (±5kbs on the island) in the single variant analysis conducted by Gaunt *et al*. (16). Therefore, these regions were analysed as before using SKAT but conditioning on the single variant responsible for the evidence of an mQTL detected at that loci. Variants which were in high linkage disequilibrium (LD) with the mQTL (defined as D’ ≥ 0.8) were removed for these regions before analysis.

Results from this analysis suggested that methylation levels were influenced independently at 3 loci by low frequency variants (MAF ≤ 5%) after conditioning analyses on reported single mQTL effects. The nearest genes to these loci were *PPP2R2A*, *C2orf80* and *SLC32A1*. All of these collapsed mQTL were acting in cis. The results for this analysis can be found in Table 5. Supplementary Material, Table S16 includes all time points in ARIES where these effects were observed to have a *P*-value < 1 × 10 ^−^ ^14^.

**Table 5.** Conditional analysis of low frequency variants collapsed by CpG island and shore regions with single variant mQTL

Conditional analysis results							**Single Variant mQTL results**	
Region	Nearest Gene	Probe	nVars05	P-value05	nVars01	P-value01	SNP	P-value
chr2:208,974,900–208,979,396	*C2orf80*	cg10392614	5	2.56x10^−20^	0	NA	rs28575061	1.36x10^−169^
chr20:37,350,130–37,359,372	*SLC32A1*	cg15490840	5	2.88x10^−20^	2	0.76	rs10932241	1.64x10^−17^
chr8:26,045,804–26,050,097	*PPP2R2A*	cg12285565	3	1.16x10^−18^	0	NA	rs2867326	1.00x10^−19^
chr16:1,003,902–1,008,281	*LMF1*	cg07338658	5	3.34x10^−13^	0	NA	rs111820009	1.06x10^−33^
chr19:41,302,467–41,307,050	*RAB4B*	cg11298343	5	6.32x10^−12^	0	NA	rs111833532	9.61x10^−50^
chr3:13,321,438–13,326,929	*NUP210*	cg05265484	3	8.54x10^−12^	0	NA	rs36024363	1.77x10^−43^
chr2:1,799,618–1,804,060	*MYT1L*	cg04722030	9	1.17x10^−11^	1	0.96	rs13387965	1.43x10^−20^
chr13:111,299,316–111,303,593	*CARS2*	cg15747390	18	1.22x10^−11^	4	0.01	rs61970542	5.87x10^−97^
chr3:112,928,437–112,933,506	*BOC*	cg23260991	3	1.56x10^−11^	3	1.56 x10^−11^	rs931702	7.48x10^−16^
chr1:91,187,139–91,191,400	*BARHL2*	cg22507154	4	1.42x10^−10^	3	9.14 x10^−7^	rs72720396	8.12x10^−25^

nVars05 = number of variants analysed (MAF ≤ 5%), *P*-value 05 = SKAT *P*-value conditioned on single variant mQTL at this loci (MAF ≤ 5%), nVars01 = number of variants analysed (MAF ≤ 1%), *P*-value01 = SKAT *P*-value conditioned on single variant mQTL at this loci (MAF ≤ 1%), SNP = mQTL variant at this loci associated with methylation from probe in single variant analysis, *P*-value = single variant *P*-value between SNP and probe from single variant analysis.

## Discussion

We have undertaken a novel approach to mQTL analysis by investigating the combined impact of multiple low frequency and rare variants on DNA methylation. Altogether we identified 95 unique regions of low frequency variants (MAF ≤ 5%) that were collectively associated with DNA methylation. 94 of these were acting in cis (associated with methylation within 1MB distance away) and 1 in trans (greater than 1MB away). Importantly, none of these effects were driven by an individual variant and therefore were not identified in the single variant analysis. Evidence of replication was observed both internally and in external datasets for the top 10 hits of this analysis which supports evidence that these associations are driven by causal effects. We also identified a further 3 loci with previous evidence of an mQTL effect from the single variant analysis, where there was evidence of an independent signal from multiple low frequency variants. This approach was less successful in identifying association signals from regions of rare variation (MAF ≤ 1%), although further studies with larger sample sizes may yield stronger evidence of association for these types of effects.

We found that 88 of the 94 unique regions responsible for observed cis-mQTL effects were identified by expanding our region of interest from CpG islands to include flanking shore regions. When conducting a variant collapsing analysis, the definition of a functional unit or genomic region by which variants are collapsed together is crucial to identifying association signals (23). This is reflected in our study, as using CpG islands alone would have overlooked the vast majority of signals identified, whereas extending regions to include both shores and shelves also resulted in fewer association signals rather than just islands and shores alone. This is most likely due to an increased number of neutral variants in the analysis window, which incorporate statistical noise into the analysis (24).

The strongest evidence of association in our study was a cis-mQTL identified near the *PFKL* gene region (*P =* 6.24×10^−24^ in the discovery analysis). Methylation of the ATF-motif in *PFKL* was observed to be reduced in obese patients compared to non-obese controls in a study investigating epigenetic modifications in terms of the aetiology of type 2 diabetes (25). *CLDN15* (*P =* 1.26×10^−20^ in the discovery analysis) was another locus which provided evidence of association in this analysis. This gene was observed to be dysregulated according to methylation status in tumour cell lines according to a recent study (26). Amongst the other top hits in our discovery analysis were mQTL identified near the *ZNF623* and *PLA2G1B* genes which have previously been reported to be hypermethylated (27,28). Previous evidence detected at these loci in methylation studies, along with the replication of these effects in external cohorts, supports the validity of the approach used in this study to detect mQTL caused by low frequency and rare variants. This is important for future studies interested in mQTL as single variant approaches may not have sufficient power to detect these types of effects.

Extending analyses to loci where there was previous evidence of an mQTL effect detected using single variant approaches identified 3 more associations between multiple low frequency variants and methylation levels. Analyses were conditioned on the previously identified mQTL, which means that this evidence suggests that these association signals are independent of each other. As with the previous analyses, applying this approach with a MAF cutoff of ≤ 1% lacked statistical power to detect any strong evidence of association. A reason for this may be due to the relatively small sample sizes for the data analysed in this study (*n = *∼800). The validity of these approaches should still be useful for future studies with larger sample sizes, in terms of detecting combined effects from rare variants on DNA methylation which would not be identified using single marker approaches. One result which was of interest involved rare variants contributing to a cis effect at the *DVL1* loci (*P =* 6.26×10^−14^), as it is a previously reported imprinted gene (29). Although evidence was not quite strong enough to survive the strict *P-*value threshold used in this study, this is encouraging for future studies which hope to detect novel variants associated with methylation by applying this approach.

Moreover, the analysis pipeline undertaken in this study can be adapted depending on the study hypothesis. For example, in this study, we have collapsed low frequency and rare variants together based on CpG island regions, although collapsing variants together across gene regions may also be a viable approach to mQTL analysis. The genotype data used in this study was imputed SNP microarray data, which is suboptimal for identifying signals from rare genetic variants. For example, on average there were 5.4 variants with a MAF less than or equal to 1% in CpG islands and flanking regions after applying appropriate quality filters. Therefore, applying this approach to next generation sequencing data which directly assays rare variants should identify evidence of an association from rare genetic variants not detected in our study.

Despite undertaking an exhaustive number of tests, we found identifying strong evidence of association for trans-mQTL challenging, an outcome also encountered by previous studies (3,30). This is likely due to smaller effect sizes relative to cis-mQTL, which is a trend also observed for trans-eQTL (31). Single variant approaches have been used to investigate the relationship between sequence variation, gene expression and DNA methylation (32). Extending the analysis framework demonstrated in this study to also incorporate the combined impact of variants on gene expression is necessary to better understand the functional consequence of rare variants. Moreover, investigating the impact of environmental exposures known to influence DNA methylation would be worthwhile to establish whether these influenced the observed associations between low frequency variants and DNA methylation. The framework easily allows for this by adjusting for covariates in the model and the findings could be important in terms of the molecular mechanisms of complex disease.

There are features of the ARIES project which should be taken into consideration when interpreting the results of this study. Firstly, all adults in the ARIES project are female and so sex was not a source of variability for the two respective time points. All methylation measurements are taken from peripheral blood, meaning we are unable to evaluate our findings using different tissue types. Furthermore, cord blood obtained from the birth time point is not equivalent to peripheral blood in its cell type composition. However, it has been reported that evidence for the majority of mQTL is consistent across tissue types (33) and the results in this study appear to reflect this as results were consistent across all time points in ARIES.

The motivation for undertaking an analysis of the association of low frequency and rare variants with DNA methylation is driven by a desire to understand the contribution made by genotype to epigenetic variation and, in turn, the role that this might play in development and disease. The biological function of the loci identified as being associated with methylation variation in this study has not been explored here but future studies are warranted.

In conclusion, we have presented a complementary approach to single variant mQTL analysis. Future studies should benefit from applying this approach as a follow-up analysis to uncover low frequency and rare variants associated with DNA methylation that may have been overlooked using single variant approaches.

## Methods

### Accessible resource for integrative epigenomic studies project (ARIES)

#### Study Sample

All samples in the discovery analysis are taken from the Avon Longitudinal Study of Parents and Children (ALSPAC)(13,14). Blood samples were taken from 1018 mother-offspring pairs (offspring at three timepoints and their mothers at two timepoints) who were enrolled as part of the Accessible Resource for Integrative Epigenomic Studies project (ARIES, http://www.ariesepigenomics.org.uk/) (12). For the purposes of the planned analyses, one timepoint in the offspring (Mean Age = 7.49 (Standard Deviation = 0.15), proportion female = 0.50), was designated for the discovery analysis, whereas measures from other timepoints were used to evaluate findings. As this data was analysed in a cross-sectional manner, adjustment for relatedness was not undertaken. Cord and peripheral blood samples were collected according to standard procedures for all available mother-offspring pairs at each time point. Written informed consent was obtained from all study participants. Ethical approval for the study was obtained from the ALSPAC Ethics and Law Committee and the Local Research Ethics Committees. Please note that the study website contains details of all the data that is available through a fully searchable data dictionary (http://www.bris.ac.uk/alspac/researchers/data-access/data-dictionary/).

#### Methylation assays

DNA samples were bisulfite treated using the Zymo EZ DNA Methylation^TM^ kit (Zymo, Irvine, CA). The Illumina HumanMethylation450 BeadChip (HM450k) was used to measure methylation across the genome and the following arrays were scanned using Illumina iScan, along with an initial quality review using GenomeStudio. A purpose-built laboratory information management system (LIMS) was responsible for generating batch variables during data generation. LIMS also reported quality control (QC) metrics for the standard probes on the HM450k for all samples and excluded those which failed QC. Data points with a read count of 0 or with low signal:noise ratio (based on a *P*-value > 0.01) were also excluded. Methylation measurements were then compared across timepoints for the same individual and with SNP-chip data (HM450k probes clustered using k-means) to identify and remove sample mismatches. All remaining data from probes was normalised with the Touleimat and Tost (34) algorithms using R with the wateRmelon package (35). This was followed by rank-normalising the data to remove outliers. Potential batch effect was removed by regressing data points on all covariates. These included the bisulfite-converted DNA (BCD) plate batch and white blood cell count which was adjusted for using the *estimateCellCounts* function in the minfi Bioconductor package (36).

#### Genotyping assays

Genotype data were available for all ALSPAC individuals enrolled in the ARIES project, which had previously undergone quality control, cleaning and imputation at the cohort level. ALSPAC offspring selected for this project had previously been genotyped using the Illumina HumanHap550 quad genome-wide SNP genotyping platform (Illumina Inc, San Diego, USA) by the Wellcome Trust Sanger Institute (WTSI, Cambridge, UK) and the Laboratory Corporation of America (LCA, Burlington, NC, USA). Samples were excluded based on incorrect sex assignment; abnormal heterozygosity (<0.320 or >0.345 for WTSI data; <0.310 or >0.330 for LCA data); high missingness (>3%); cryptic relatedness (>10% identity by descent) and non-European ancestry (detected by multidimensional scaling analysis). After QC, 500,527 SNP loci were available for the directly genotype dataset. Data for ALSPAC mothers was generated using the Illumina human660W-quad genome-wide SNP genotyping platform (Illumina Inc, San Diego, USA) at the Centre National de Génotypage (CNG, Paris, France). Samples were excluded due to non-European ancestry, missingness, relatedness, heterozygosity or gender mismatches. PLINK (v1.07) (21) was used for QC on an initial set of 10,015 subjects and 557,124 directly genotyped loci. Following QC the final directly genotyped dataset contained 526,688 SNP loci.

Imputation was performed for all genotyped mothers and children to improve SNP density. ShapeIt (version 2 revision 727) was used to phase genotypes and Impute (version 2.2.2) was used to impute this data using the 1000 genomes reference panel (phase 1 version 3, phased using ShapeIt version 2, December 2013, using all populations). Genotypes were then filtered to include those with a Hardy-Weinberg equilibrium of *P *> 5 × 10 ^−^ ^7^, MAF ≤ 5% and imputation info score > 0.8. The final imputed dataset for all subsequent analyses contained 3,721,682 loci. 1,787,681 of these had a MAF ≤ 1%.

### Replication cohorts

#### TwinsUK

The TwinsUK cohort was established in 1992 to recruit monozygotic and dizygotic twins (20). More than 80% of participants are healthy female Caucasians (age range from 16 to 98 years old). The cohort includes more than 13,000 twin participants from all regions across the United Kingdom, and many have had multiple visits over the years. The TwinsUK cohort has been used in many epidemiological studies and is representative of the general UK population for a wide range of diseases and traits (37).

Samples from TwinsUK were genotyped using the Illumina Hap317K and Hap610K assays (Illumina, San Diego, USA) following standard procedures. Normalised intensity data were pooled and genotypes called on the basis of the Illluminus algorithm (38). No calls were assigned if the most likely call was less than a posterior probability of 0.95. SNPs that had a low call rate (<95%), Hardy-Weinberg *P* values < 10 − 4 were excluded. We also removed subjects if the sample call rate was less than 95%, autosomal heterozygosity was outside the expected range, genotype concordance was over 97% with another sample and the sample was of lesser call rate. Imputation of genotypes was carried out using the software IMPUTE (39).

DNA methylation was measured for 877 individuals randomly selected from the TwinsUK cohort, 847 who also had genetic information. The Infinium HumanMethylation450 BeadChip (Illumina Inc, San Diego, CA, USA) was used to measure DNA methylation. Details of experimental approaches have been previously described (40). Normalization was carried out using the ‘minfi’ R package (41), a procedure equivalent to the Lumi:QN + BMIQ pipeline. DNA methylation probes that mapped incorrectly or to multiple locations in the reference sequence and probes with a detection *P* value of >0.05 or missing values were removed, resulting in 452,874 probes. Blood cell type coefficients were estimated from the methylation data using the method described by Houseman *et al.* (42). For this project, normalized methylation beta values were regressed out effects of family structure, batch effects and predicted cell count data. The obtained methylation residuals were used to test the association between genetic variants and methylation.

#### Exeter

These samples are the first phase of a multi-stage case-control EWAS of schizophrenia (Hannon *et al.* 2016. Submitted). 500ng of DNA from each sample was treated with sodium bisulfite using the EZ-96 DNA Methylation kit (Zymo Research, CA, USA). DNA methylation was quantified using the Illumina Infinium HumanMethylation450 BeadChip (Illumina Inc, CA, USA) run on an Illumina iScan System (Illumina, CA, USA) following a standard protocol. Samples were randomly assigned to chips and plates to ensure equal distribution of cases and controls across arrays and minimise batch effects. Data were imported in R programming environment using the *methylumIDAT*() function in the methylumi package (43). Our stringent quality control pipeline included checking methylated and unmethylated signal intensities, bisulfite conversion efficacy, tissue prediction (of blood origin) from the Epigenetic Clock software (https://dnamage.genetics.ucla.edu/) (44), gender and detection p values of all samples. Principal component (PC) analysis was used (calculated across all probes) to identify outliers, excluding samples > 2 standard deviations from the mean for both PC1 and PC2. Normalization of the DNA methylation data was performed used the *dasen*() function in the wateRmelon package (35). Genotyping was performed using the Affymetrix Mapping 500K Array and the Genomewide Human SNP Array 5.0 or 6.0 (Affymetrix, CA, USA). Genotypes were culled from raw intensity data using the Birdseed component of the Birdsuite algorithm (45,46). Samples were genotyped by the Genetic Analysis Platform at The Broad Institute of Harvard and MIT according to standard protocols. All samples were concordant across the methylation and genotype data for SNPs assayed on both platforms. Prior to imputation, PLINK (21) was used to remove samples with >5% missing data. We also excluded SNPs characterized by >5% missing values, a Hardy-Weinberg equilibrium *P*-value < 0.001 and a minor allele frequency of <5%. Imputation was performed using ChunkChromosome (http://genome.sph.umich.edu/wiki/ChunkChromosome) and Minimac2 (47,48) with the 1000 Genomes reference panel of European samples (phase 1, version 3). Imputed genotypes were then converted back in the PLINK format files using GCTA software (49) only including variants with Rsq > 0.1.

### The UK10K project

DNA Samples from 4,030 UK10K study participants (2,040 offspring from the ALSPAC cohort, 1,990 from the TwinsUK cohort) were subjected to low coverage (6-8x average read depth) whole-genome sequencing (WGS). Sequencing was performed at both the Wellcome Trust Sanger Institute (WTSI) and the Beijing Genomics Institute (BGI). DNA (1-3μg) was sheared to 100–1000 bp using a Covaris E210 or LE220 (Covaris, Woburn, MA, USA). Sheared DNA was size subjected to Illumina paired-end DNA library preparation. Following size selection (300–500 bp insert size), DNA libraries were sequenced using the Illumina HiSeq platform as paired-end 100 base reads according to manufacturer’s protocol.

Data that passed quality control (QC) was aligned to the GRCh37 human reference used in phase 1 of the 1000 Genomes Project. Reads were aligned using BWA (v0.5.9-r16) (50). Of the 4,030 participants, 3,910 samples (1,976 ALSPAC and 1,934 TwinsUK) went through the variant calling procedure. Low quality samples were identified by comparing the samples to their GWAS genotypes using about 20,000 sites on chromosome 20. A total of 112 samples (48 ALSPAC and 64 TwinsUK) were removed, leaving 3,798 samples (1,928 ALSPAC and 1,870 TwinsUK) that were eligible for the genotype refinement phase.

Missing and low-confidence genotypes in the filtered VCFs were refined out using the imputation procedure in BEAGLE 4 (51) with default parameters. Additional sample-level QC steps were carried out on refined genotypes, resulting in 17 samples (16 TwinsUK and 1 ALSPAC) being removed due to either non-reference discordance with GWAS SNV data > 5%, multiple relations to other samples or failed sex check. A principal components analysis was conducted using EIGENSTRAT (52) to exclude participants of non-European ancestry after merging our data with a pruned 11 HapMap3 population dataset (53). 44 subjects (12 TwinsUK and 32 ALSPAC) did not cluster to the European (CEU) cluster and were removed. The final sample size for association analyses comprised of 3,621 individuals (1,754 TwinsUK and 1,867 ALSPAC).

### Statistical analysis

#### Discovering novel mQTL

Annotations for UCSC CpG Islands were obtained using the R Package ‘COHCAPanno’(15) according to the hg19 build of the human reference genome. All low frequency variants (MAF ≤ 5%) were collapsed together within regions where there were no mQTL loci identified from the individual variant analysis carried out by Gaunt *et al*. (16) using the same dataset. These regions were defined as:

1. CpG islands as defined by UCSC co-ordinates
2. CpG islands and shores (i.e. islands +2kbs up and downstream)
3. CpG islands, shores and shelves (i.e. islands +4kbs up and downstream).

#### Cis-mQTL

Regions which had a reported mQTL in close proximity (CpG island ± 5kb) were not analysed here, but in a subsequent analyses conditioning on the reported mQTL effect. The remaining regions which had least 2 variants were analysed using the Sequence Kernel Association Test (SKAT) (18) with each CpG probe in turn that was within +/- 1Mb distance from the region analysed (30). Regions with only 1 variant were not evaluated as there was no benefit to applying a collapsing method in these instances. Other types of collapsing methods make assumptions about the direction of effect for variants within the analysis window. As we hypothesised that variants collapsed across these regions may have conflicting directions of effects (i.e. variants within a region may cause methylation levels to either increase or decrease at a particular loci), SKAT was chosen above alternatives. Details on SKAT can be found in the publication by Wu *et al*. (18). In brief, SKAT uses a linear model in this study as our outcome of interest is continuous:

$$y_{i}=\alpha_{0}+\;\alpha \prime X_{i}+\;\beta \prime G_{i}+\epsilon_{i}$$

where y is the rank normalized measure of DNA methylation, α_0_ is the intercept term, [α_1_,…,α_m_]’ is a vector of regression coefficients for m covariates, X_i_ = [X_i1_,…,X_im_] denotes covariates, β = [β1,…,βp]’ is the vector of regression coefficients for the p observed variants with a region, G_i_ = [G_i1_,…,G_ip_] denotes the genotypes for p variants within the region (i.e. 0, 1 or 2) and ϵ is the error term. SKAT assumes that the genetic effect β_j_ of an individual variants j follows an arbitrary distribution with mean 0 and variant w_j_τ_i_ where τ is a variance component and w_j_ is a weight of variant j. SKAT assumes that $\sqrt{w}$_j_ follows a Beta(MAF_j_; a_1_, a_2_).

This analysis was undertaken using two MAF cut offs of ≤ 5% (for low frequency variants) and ≤ 1% (for rare variants). We used a conservative multiple testing correction of P < 1.0x10 ^−^ ^14^ as undertaken by Gaunt *et al.* (16). This was to reduce the number of false positive findings. Individual variant effects from regions that survived this correction were evaluated using linear regression to ensure that no individual variant would have been identified in the previous study, but when analysed together with other low frequency or rare variants we observed much stronger evidence that they were collectively influencing methylation.

#### Trans-mQTL

We applied the same approach as above but to identify trans-mQTL (defined as associations between variants and CpG probes more than +/- 1Mb from regions analysed). Due to the computational demand required for the number of tests, we collapsed variants together only using CpG islands with flanking shore regions, as these regions provided the number of association signals that survived the correction for multiple comparisons in the cis-analysis. This analysis was undertaken using 2 MAF cutoffs of 5% and 1% and evaluated with the same multiple testing correction as before.

#### Analyses in other time points

Analyses were initially undertaken using all available individuals from the Childhood time point in ARIES (Mean Age = 7.49 (Standard Deviation = 0.15), proportion female = 0.50), which was designated as our discovery analysis. Results which survived the correction for multiple testing were further evaluated by analysing the same set of variants with methylation values measured at the same probe from all other time points in both children and mothers. These analyses were conducted using each time point in turn and without adjustment for relatedness. Variants which did not exist amongst the sample of mothers were not replaced in these analyses (i.e. we attempted to replicate the effects with available variants without replacement). We did not evaluate all potential cis- and trans-mQTL at all-time points due to the computationally exhaustive number of analyses needed, as well as the potential number of false positive findings incurred by doing so. Evidence that hits replicated at other time points was based on associations with a lower threshold p-value of *P >* 1 × 10^−7^ on the basis that these results are supported by their combination with other evidence from time points through the life course.

#### Replication in independent cohorts

Replication analyses were conducted using SKAT to evaluate the association between sets of variants and DNA methylation using the same 450k probe ID. Variants which were not eligible or available within the replication cohorts were not included in replication analyses.

#### Leave-one-out analysis

We firstly generated box and whisker plots for the top 10 hits of our analysis to discern whether extreme measures of DNA methylation were influencing our results. A leave one out analysis was also undertaken on the top 10 hits to further ensure that our results were not heavily influenced by potential outliers or individual effects. The purpose of this analysis was also to illustrate the combined effect of these sets of variants on methylation. Variants within regions were first of all pruned using plink software (21), followed by re-running our analysis for each mQTL except systematically removing individual variants in turn. Results were plotted with –log10 *P*-values on the y-axis and the variant removed on the x-axis. Plots were annotated with red lines to show the observed *P*-value when all variants were analysed (prior to pruning). A blue line was also added to show the *P*-value threshold used in our study (i.e. *P* < 1.0 x 10 ^−^ ^14^).

#### Whole genome sequence evaluation

To evaluate whether overlapping rare variants in the probe sequence was incorporating bias into our analysis, we took a subset of individuals from the discovery analysis who had whole genome sequence (WGS) data from the UK10K project (22). This was due to the fact that Naeem *et al*. had potentially not evaluated these variants in their study when looking at overlapping SNPs (17). Using the top 10 hits identified in our study, corresponding probe locations were identified to verify whether there was an overlapping variants in the WGS data. When this was the case, a conditional analysis was undertaken using individuals enrolled in both the ARIES project and UK10K. This analysis involved repeating the collapsed mQTL analysis as before except including the overlapping variant in the probe sequence as a covariate in the model. An attenuation in *P*-value would indicate that our analysis may be influenced by the overlapping rare variant in probe sequence.

#### Conditional analysis for regions with a single variant mQTL

For CpG island regions where an mQTL was identified in the previously undertaken single variant analysis, we undertook conditional analyses to evaluate whether there was an independent effect from regions of low frequency and rare variants at these loci. All CpG island regions not included in the previous analysis were eligible. mQTL results from the single variant analysis had been previously analysed with GCTA (49) to identify independent loci associated with each methylation probe.

Low frequency variants (MAF ≤ 5%) were collapsed as before within CpG islands and their flanking shore regions. Variants which were in high linkage disequilibrium (LD) with the mQTL (defined as D’ ≥ 0.8) were removed for these regions. *r*^2^ values for LD were not used as the range of *r*^2^ is dependent on allele frequencies, which could potentially be very different between a common SNP and rare genetic variants. Regions with at least two variants remaining were analysed using SKAT with the corresponding probe, which was associated with the single variant mQTL at this site. The SNP responsible for the mQTL was included as a covariate in the model. Analyses were run using two MAF cutoffs of 5% and 1% as before for the collapsed regions of low frequency variants. *P*-values lower than 1×10^−14^ were analysed a further time but including a covariate matrix consisting of all SNPs responsible for an mQTL effect at this locus. This was to ensure results were not tagging a different mQTL signal not accounted for in the initial analysis. Analyses were undertaken using all time points in ARIES. All statistical analyses were undertaken using R statistical software (54).

## Supplementary Material

Supplementary Material is available at *HMG* online.
